# Peptide-Based Functional Biomaterials for Soft-Tissue Repair

**DOI:** 10.3389/fbioe.2019.00205

**Published:** 2019-08-23

**Authors:** Katsuhiro Hosoyama, Caitlin Lazurko, Marcelo Muñoz, Christopher D. McTiernan, Emilio I. Alarcon

**Affiliations:** ^1^Division of Cardiac Surgery Research, University of Ottawa Heart Institute, Ottawa, ON, Canada; ^2^Biochemistry, Microbiology and Immunology Department, Faculty of Medicine, University of Ottawa, Ottawa, ON, Canada

**Keywords:** peptides, biomaterials, tissue engineering, functional materials, synthetic polymers

## Abstract

Synthetically derived peptide-based biomaterials are in many instances capable of mimicking the structure and function of their full-length endogenous counterparts. Combine this with the fact that short mimetic peptides are easier to produce when compared to full length proteins, show enhanced processability and ease of modification, and have the ability to be prepared under well-defined and controlled conditions; it becomes obvious why there has been a recent push to develop regenerative biomaterials from these molecules. There is increasing evidence that the incorporation of peptides within regenerative scaffolds can result in the generation of structural recognition motifs that can enhance cell attachment or induce cell signaling pathways, improving cell infiltration or promote a variety of other modulatory biochemical responses. By highlighting the current approaches in the design and application of short mimetic peptides, we hope to demonstrate their potential in soft-tissue healing while at the same time drawing attention to the advances made to date and the problems which need to be overcome to advance these materials to the clinic for applications in heart, skin, and cornea repair.

## Introduction

Bioinspired materials for tissue repair have been amongst the most exhaustively explored fields in biomaterials research, yet mimicry of native extra cellular matrix (ECM), remains one of the most challenging tasks in tissue engineering. Further, while remarkable progress in recombinant protein expression has been made, there remains a gap as these processes are still relatively expensive particularly for heteromeric proteins; thus limiting scientists to the use animal origin (e.g., extracted from tissues) proteins including collagen and elastin for engineering translatable biomaterials. This limitation has severely halted clinical translation of functional biomaterials to the clinic. It is well-known that proteins take on an important role in almost all biological processes. While they have well-defined roles in the structural integrity of cells, organs, and tissues; their roles in other processes such as cell motility, signal transduction, immunological response, and enzymatic reactions are much more dynamic (Ouzounis et al., 2003). As such, many of the important findings regarding wound healing and tissue repair have come through the study of protein-protein and protein-ligand interactions. One such finding is that molecules which present binding sites for proteins, typically associated with either disease or wound healing, are excellent targets for the development of therapeutic solutions (Webber et al., 2010b). Given recent advancements in both chemical peptide synthesis and the recombinant production of full length proteins and peptides, generating and studying the interactions of mimetic molecules has been greatly simplified. Whether it is the production of exact copies of full-length or fragments of proteins, the incorporation of non-coded amino acids, or modification of the peptide backbone to enhance its proteolytic stability or the inclusion of tethers for further functionalization; one can generally find a suitable method to produce the desired molecule. For these reasons peptides, which are the small building blocks of proteins, have rapidly emerged as a cost-effective alternative for developing functional materials for tissue repair.

While the design options are almost limitless, these mimics usually interact with their target through the presentation of a specific amino acid sequence, a functional structure, or a combination of both. In this review, we will focus on peptides structures prepared using solid-phase peptide synthesis (SPPS), which for most systems nowadays takes place in cyclically automated synthetic equipment where each amino acid of the peptide structure is sequentially incorporated. Readers interested in learning more on peptide synthesis using transgenic organisms are encouraged to seek out reviews on this specific topic (Structural Genomics et al., 2008).

SPPS was conceptualized in 1959 and first reported on in the early 1960's by the Nobel awardee Robert Bruce Merrifield, its popularity and mainstream adoption grew in the 1970's and 1980's as technological advances in peptide chemistry made the process more robust (Merrifield, 1963). The concept of extending a peptide chain through the α-Nitrogen of an amino acid (*a.a*.) by coupling it with the carboxyl terminal of the next sequential *a.a*. whose other functional groups are protected, truly revolutionized the way peptides chains were produced. These protecting groups serve to prevent both oxidations and unspecific reactions of the *a.a*. side chains (Isidro-Llobet et al., 2009). Decades of intense synthetic research have yielded a number of versatile protecting groups such as the archetypical Fluorenylmethyloxycarbonyl (Fmoc) group. Single *a.a*.'s bearing Fmoc group are readily available on the market (Sigma-Aldrich, 2019). Technological improvement in SPPS, such as the use of microwave reactors and advanced temperature control systems, has allowed for the synthesis of peptides containing hundreds of *a.a*.'s in improved yield and reaction time in comparison to room temperature and convectional heating methods (Bacsa et al., 2006; Loffredo et al., 2009; Pedersen et al., 2012; Thapa et al., 2014). These technological advances have also contributed to the expedited synthesis of so-called difficult peptide sequences. Difficult peptide sequences refer to peptide sequences that agglomerate forming insoluble products during synthesis or after removal of the protective groups, a process that results in reduced yields or deactivation of the peptide preventing further modifications (Tickler and Wade, 2007). In most instance these problems arise due to introduction of functionalities capable a participating in non-covalent interactions, such as hydrogen bonds and dipole-dipole interactions (Paradis-Bas et al., 2016). Thus, when designing peptides for SPPS, both the individual *a.a*. and the resulting coupling products (on the resin) should be screened for the potential formation of self-assembled structures, side reactions, and tendency to fold onto the resin. The following parameters have been demonstrated to aid in the synthesis of difficult peptide sequences: (i) high temperatures (e.g., 95°C for microwave-assisted synthesis), (ii) presence of salt or detergents for improving solubility, (iii) protecting groups at the amide group to avoid potential hydrogen-bond interactions, (iv) incorporation of amino acids with unreactive side chains to prevent undesired interactions, and (v) glycosylation or pegylation to improve peptide solubility.

## Bioactive Peptide Sequence Mimics

**Scheme 1:**
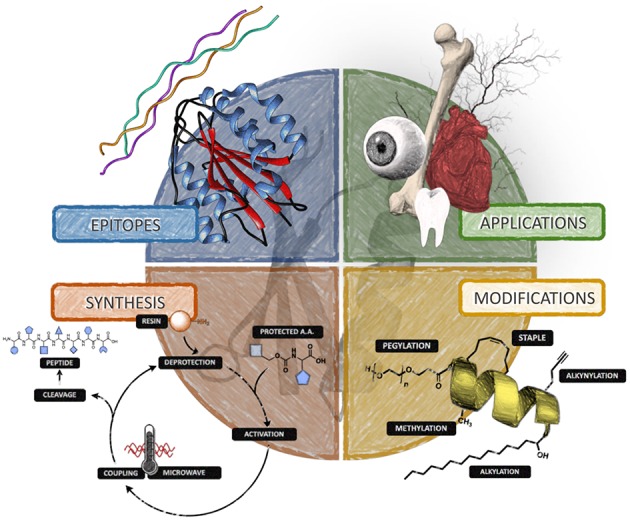
Diagram summarizing the main concepts revised in this review for the use of peptide-based materials in tissue and organ repair.

Structural mimics developed using peptide sequences are in fact epitopes of bioactive sites, where in many instances the recognition site of the mimic is defined by both the amino acid sequence and three-dimensional conformation. In the following sections, we will discuss a number of bioactive short peptide sequences that have been identified, synthesized, and/or incorporated into structures to impart some type of biological response which could be exploited in tissue engineering or the development of regenerative biomaterials. The peptide sequences which will be discussed correspond to a representative set of examples, which we believe fall into one of the following three categories which we deemed most important in the development of functional biomaterials for the regeneration of heart, skin, and corneal tissue, namely, (i) pro-angiogenic sequences, (ii) anti-inflammatory, and (iii) pro-adherence sequences. Table 1 contains some representative peptide sequences that have been identified and used in the fabrication of functional materials. Scheme 1 depicts a representative summary for the concepts revised in this review. While we have limited this review to our expertise in soft tissue targets such as the heart, skin, and cornea, it is important to mention that peptide based materials have also found applications in the regeneration of hard-tissues such as bone and teeth, and that further information regarding these applications and recent advancements can be found in more specialized reviews (Pountos et al., 2016; Wang et al., 2017).

**Table 1:** Representative peptide sequences of potential interest in the development of functional biomaterials for tissue engineering.

	**Peptide Sequence**	**Reference(s)**	**Main findings**	**Limitations**	**Portion of protein extracted**	**Receptors involved**
**ECM PROTEINS**						
Collagen	GFOGER	Knight et al., 2000; Wojtowicz et al., 2010	Authors demonstrate the utility of coating grafts and improvement in bone growth with the peptide	Used an original sequence that included a GGYGG sequence that does not demonstrate utility in the self-assemble process and was added originally for radiolabeling (Reyes and Garcia, 2003)	Residues 502–507 of the αl(I)-CB3 fragment of type I collagen	α2β1 integrin receptor
	DGEA	Mehta et al., 2015	DGEA induced osteogenesis only when encapsulated with cells in a 3D network. The peptide does not provide any advantages when used in 2D cell culture	The authors do not provide details regarding the nature of peptide attachment to the polymer or whether it formed dimers through the carboxylic acids of the peptides	Residues 435–438 of the αl(I)-CB3 fragment of type I collagen	α2β1 integrin receptor
	FPGERGVEGPGP	Gelain et al., 2006; Bradshaw et al., 2014	Authors demonstrated the ability of the peptide to induce migration of fibroblast in hydrogels	The peptide itself does not induce cell proliferation	–	–
Laminin	IKVAV	Tashiro et al., 1989; Yamada et al., 2002	Authors demonstrate that the peptide promotes cell attachment	The peptide sequence does not produce the same response as laminin	From the α-helix A chain segment of fragment E8 starts at amino acid position 1886	–
	YIGSR	Graf et al., 1987b; Yoshida et al., 1999; Boateng et al., 2005	They demonstrated the ability of the peptide to attach cardiomyocytes onto a silica treated surface	The peptide sequence does not produce the same response as laminin	β1 chain amino acid residues 929–933 on the of Laminin-1	–
	PDGSR	Kleinman et al., 1989; Huettner et al., 2018	They demonstrated an improvement in the adhesion of tumoral cells in the presence of the peptide	They compared the peptide with a cyclic YIGSR peptide, but did not provide information if the cyclic PDGSR could be improved too	β1 chain amino acid residues 902–906 on the of Laminin-1	–
	LRE	Hunter et al., 1991	They assessed the active protein recognition of the peptide for neurite outgrowth and its correlation with salts in the solution	No direct assessment of antibody interaction to identify the specific receptor involved in the interaction	The A-subunit of laminin and synaptic basal lamina	–
	IKLLI	Tashiro et al., 1999	They demonstrated attachment of cells is similar to that seen with IKVAV peptide. Also demonstrate that conformation of the peptide in a secondary structure affects adhesion	They did not use other highly charged positive peptides to compare the affinity of the heparin	α1 chain of laminin, between amino acids residues 2080–2095	Integrin receptor α3β1 and a cell surface heparan sulfate proteoglycan
Fibronectin	RGDS	(Ruoslahti, 1988; D'souza et al., 1991; Leahy et al., 1996)	One of the most used sequences for cell attachment	It is not the only site involved in cell attachment and recognition, other molecules could be also key, as an example proteoglycans.	Domain 10, from amino acid sequence 1493 to 1496	More than 10 RGD dependent receptors, as an example: α3β1, α5β1, αvβ1, etc…
	KQAGDV	Hautanen et al., 1989; Calvete et al., 1992	They provide well-documented information of attachment sectors of the peptide to the receptor	The authors did not show inhibition studies with the peptides under study	γ chain in the fibronectin protein	αIIbβ3, αVβ3
	REDV	Hubbell et al., 1991; Massia and Hubbell, 1992	Demonstrate similarities between this peptide and RGD peptide, also selectivity for vessel forming endothelial cells	–	Within the spliced type III connecting segment (III CS) domain of human plasma fibronectin	α4β1
	PHSRN	Feng and Mrksich, 2004	Demonstrate that this fragment is also recognized by integrin receptor, competitive with RGD, but with less strength than RGD	–	Within the 9th type III domain	α5β1
**REMODELING ENZYMES**						
Collagenase	GPQGIWGQ GPQGYIAGQ GPQGYILGQ	Nagase and Fields, 1996; Lutolf et al., 2003; Patterson and Hubbell, 2010	Substrates containing the sequence are cleaved under the conditions tested and can induce release of specific molecules after proteolytic effects	The sequence is not specific for one type of enzyme	Sequence presented in position 775 of αI fibril of the collagen	GPQG↓ (↓ = Enzyme proteolytic effect)
Matrix metalloproteinases (MMPs)	CPENYFFWGGGG	Salinas and Anseth, 2008	Demonstrate that biomaterials performance depends on the presence and dynamic concentration of the receptor in the hydrogel	In hydrogels, the enzyme degradation rate is fast for surface and slow for deeper cues	Cleaved by MMP-13	CPEN↓
	APGL	West and Hubbell, 1999	The sequence is selective for collagenase, but not for plasmin.	The authors do not provide proof the sequence could be cleaved through cell culture	Cleaved by collagenase	APG↓
	LGPA	Patel et al., 2005	The sequence is attached to a photo responsive material, that can control hydrogel formation with light and degradation by the peptide sequence.	The sequence by itself does not induce cell attachment and survival in the long term	Collagenase-sensitive degradable sequence	–
	GTAGLIGQ	Jun et al., 2005; Kim et al., 2009	The sequence is used to release other drugs, in this case cis-platin	The sequence is attached with an RGD sequence. This could affect enzymatic degradation rates (not evaluated without the RGD sequence)	MMP-2 specific cleavage	GTAG↓
Plasmin	YKNRD	Pratt et al., 2004; Raeber et al., 2005	The sequence induces bone regeneration and cell attachment. Selective to plasmin	–	Plasmin sensitive sequence that is enhanced at the carboxylic side of the lysine amino acid	YK↓
	ELAPLRAP FPLRMRDW EGTKKGHK KKGHKLHL HPVGLLAR	Patterson and Hubbell, 2011; Singh et al., 2012	Depending on the sequence selected, the hydrogel degradation rate can be tuned with respect to its sensibility toward plasmin	Sequences have shared activity with other MMPs	–	ELAP↓ FPLR↓ EGTKKGHK↓ KKGHK↓ HPVG↓
**TARGET PROTEIN/RECEPTOR**						
Vascular endothelial growth factor	KLTWQELYQLKYKGI	Diana et al., 2011; Liu et al., 2012	Demonstrated ability to promote angiogenesis		VEGF mimetic peptide agonist from amino acid sequence 87 to 100	VEGF receptor 1-D2
	LRK_2_LGKA	Webber et al., 2010a	Cationic amino acids are used to bind heparin binding factors to a self-assembling sequence	The attachment of the heparin binding is ionic and is not compared with covalent bonding, which could increase long term release of the factors	–	–
Glycosaminoglycans	PNDRRR	Gilmore et al., 2013	Heparin binding through the sequence RRR (or KKK) is used for increasing angiogenic properties	The attachment of heparin is ionic, thus reducing the long-term stability of the aminoglycan	–	–
**SUPRAMOLECULAR STRUCTURE**						
Vesicle/Micelle	G_4_D_2_ G_6_D_2_ G_8_D_2_ G_10_D_2_	Santoso et al., 2002	Length of the peptide glycine chain, dictated the formation of nanovesicles or nanotubes	Lack of homogeneous structures	–	–
	V_6_K_2_ L_6_K_2_ A_6_K V_6_H V_6_K H_2_V_6_ KV_6_	Von Maltzahn et al., 2003	The peptides have the ability to self-assemble in different macro-structures. One of the main advantages, is that they dissemble above their pI	Lack of homogeneous structures	–	–
Fiber	(PKG)_4_(POG)_4_(DOG)_4_	O'leary et al., 2011	Stable formation of a hydrogel that has similar characteristics to collagen	Lack of D periodicity	–	–
	PRG)_4_(POG)_4_(EOG)_4_	Rele et al., 2007	Stable formation of a hydrogel that has similar characteristics to collagen	Lack of strength when compared to collagen bundles	–	–
	(RADA)_4_ (RARADADA)_2_ (FKFE)_2_ (KLDL)_3_	Sieminski et al., 2008	Fibers are formed by β-sheet interactions. RADA incorporation leads to better attachment of cells	Ability to control fiber dimensions could improve comparison of the system	–	–
**MULTI-DOMAIN PEPTIDES**						
Double function peptide	E_2_(SL)_6_E_2_-G-RGDS	Bakota et al., 2011	Left sequence used for self-assembly as a β-sheet [E_2_(SL)_6_E_2_]. Right sequence to be sensed as fibronectin receptor [RGDS]	–	–	–
	C_12_H_25_O-YGAAKKAAKAAKKAAKAA	Chu-Kung et al., 2004	Left sequence: lipid portion to interact with lipidic membranes [C_12_H_25_O]. Right sequence: cationic sequence to facilitate interaction with bacteria wall as an anti-microbial peptide [YGAAKKAAKAAKKAAKAA]	Lipid attachment could result in toxicity toward eukaryotic cells	–	–
Quadruple function peptide	KS(LS)_2_-LRG-(SL)_3_KG- KLTWQELYQLKYKGI	Kumar et al., 2015	Left sequence [KS(LS)_2_] used for self-assembly as a β-sheet Center left sequence (LRG) MMP-2 substrate. Center Right sequence [(SL)_3_KG]: used for self-assembly as a β-sheet Right sequence [KLTWQELYQLKYKGI] is a vascular endothelial growth factor	–	–	–

### Pro-Angiogenic Sequences

**Figure 1:**
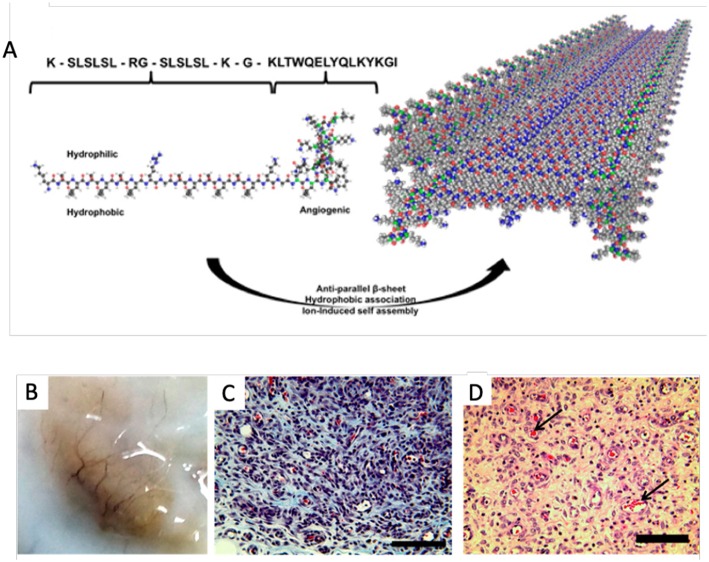
Self-assembling angiogenic peptide hydrogel. **(A)** Schematic illustrating the structure of the the multi-domain peptide comprising the VEGF mimic QK sequence and its assembly into a β-sheet. **(B)** Visible macroscale vessels apparent within the explant materal 7 days post injection. **(C)** Massons's Trichrome and **(D)** HandE staining showing infiltration of scaffolds and presence of blood vessels with red blood cells [arrows] at 1 week post injection; scale bar 100 μm. Adapted with permission from Kumar et al. (2015). Copyright 2015 American Chemical Society.

Angiogenesis is a process which involves the proliferation and migration of endothelial cells as well as the concurrent remodeling of the extracellular matrix, which drives the development of new blood vessels from exiting vasculature (Potente et al., 2011). The principal regulator of angiogenesis in both physiological and diseased state is vascular endothelial growth factor (VEGF). VEGF and its many isoforms induce physiological responses through interaction with three well-described tyrosine kinase receptors (Simons et al., 2016). To date the most promising pro-angiogenic peptides are VEGF mimics. Of the available VEGF-mimics the most well-characterized is peptide QK, which was designed to imitate the binding of VEGF to its receptor through a N-terminal α-helix mimic comprised of the amino acid sequence, KLTWQELYQLKYKGI (Andrea et al., 2005). The angiogenic properties of peptide QK have been demonstrated both *in vitro* and *in vivo*, with evident endothelial cell activation and increases in VEGF related cellular functions such as chemotaxis, invasion, sprouting of new capillaries, and enhanced organization (Andrea et al., 2005; Finetti et al., 2012). A self-assembling β-sheet peptide hydrogel encompassing the QK sequence has also shown to promote cell infiltration and vascularization when injected subcutaneously in a rat model as shown in Figure 1 (Kumar et al., 2015).

It may also be interesting to explore the ability of these VEGF mimics to bind heparin as there is much literature regarding the propensity of different isoforms of VEGF to bind heparin and the necessity of this binding to promote endothelial cell growth and proliferation (Ferrara et al., 2003). Furthermore the incorporation of VEGF within oxygen generating or hypoxia inducing hydrogels or matrices could potentially influence hypoxia inducible factors which are important in the expression and function of VEGF (Krock et al., 2011).

Other peptides which have shown promise in regulating angiogenesis are targets of growth factor receptors which typically work in conjunction with VEGF. For example, fibroblast growth factor as well as neural cell adhesion molecules (NCAMs), which have been shown to bind to fibroblast growth factor receptors can also promote angiogenesis (Elfenbein et al., 2007). Peptide mimics of both FGF2 and NCAM have been prepared synthetically and while they may act in either a canonical or non-canonical fashion, there is strong evidence that they influence angiogenesis (Elfenbein et al., 2007; Rubert Pérez et al., 2017). There are also a number of angiopoietin-1 mimics which have shown promise in regulating angiogenesis via interaction with a tyrosine kinase receptor (Tie2) which is found primarily on vascular endothelial cells and hematopoietic cells (Cho et al., 2004; Miklas et al., 2013). Other peptides one may want to incorporate within materials destined for vascularized tissue are those capable of mimicking transforming growth factors (TGFα and TGFβ) (Ferrari et al., 2009), tumor necrosis factor (TNFα) (Sainson et al., 2008), Angiogenin (Hu et al., 1997), Interleukin 8 (IL8) (Li et al., 2008), or hepatocyte growth factor (HGF) (Xin et al., 2001) as these mitogens and chemokines have been demonstrated to promote angiogenesis through control of endothelial cell growth and/or interaction with VEGF mediated pathways. Considering the effect these factors can have on the expression and efficacy of VEGF and that they are typically targets of surface bound receptors, their incorporation into soft materials may need to be done in such a way that their interaction with the target receptor is not hindered, which may limit covalent attachment within the matrix.

### Anti-inflammatory Sequences

In the design of scaffolds and biomaterials for tissue engineering and regeneration, the host immune system is one of the largest barriers to overcome. However, this does not mean that immune response is to be completely avoided; in fact in order to maximize the therapeutic efficacy of implants it is necessary for them to modulate the resulting immune response. Furthermore, inflammation promotes angiogenesis and the formation of new blood vessels can lead to further inflammation. For this reason it is important to understand that inflammation is a complex process which eventually brings homeostasis to the effected tissue through the promotion of cell infiltration, proliferation, and subsequent polarization. While there are many players involved in immune response, macrophages are considered amongst the most important and as such the current section will focus on the ways peptide mimetics can or could be used in their regulation. Macrophages are dynamic cells whose phenotype is subject to polarization from the extracellular environment as well as active signaling molecules (Taraballi et al., 2018). Classically, when discussing the phenotype of macrophages, there is said to be two distinct subsets (i) M1 (pro-inflammatory) and (ii) M2 (anti-inflammatory/pro healing). However, this is a highly simplified view considering the polarization toward either phenotype is actually more of a continuum with the difference between M1 and M2 not being discrete (Martinez and Gordon, 2014). Through the design of short peptides that interact with immunogenic receptors of M2 macrophages like TGF-bR, IL-4R, IL-6R, IL-10R, and MCSFR it is possible to modulate the immunological response associated with tissue damage and repair as well as the introduction of foreign materials (Taraballi et al., 2018). Upon acting on the expression of both pro- and anti-inflammatory cytokines such IL 6 and TNF-α as well as the production of reactive oxygen species one could develop materials which can reduce inflammation, recruit cells via chemotaxis, and ultimately improve wound healing (Boersema et al., 2016). While most inflammation related to foreign material response can be eliminated or reduced through the use of recombinant or autologous proteins or protein/peptide mimics, it may be possible to include small peptide mimetics to activate and polarize macrophages toward a type 2 phenotype. However, due to the complexity of the activation process it is difficult to pin down a sequence or multiple sequences which could bring about the desired response; and for this reason there are not many sequences known to modulate immune response in ways which are beneficial to tissue regeneration and the design of regenerative biomaterials. There are also a number of sequences defined as being anti-bacterial and as such anti-inflammatory (Rotem and Mor, 2009). One class of peptides which have shown promise in modulating immune responses are innate defense regulator (IDR) peptides (Niyonsaba et al., 2013). These cationic antimicrobial peptides are synthetic cationic analogs of naturally occurring host defense peptides or proteins (HDP). They are relatively short peptides (10–50 *a.a*.), with no specific consensus sequence. While they have some ability to directly kill microbes, they are also capable of modulating immune and inflammatory responses. For example, they are capable of influencing chemotaxis, stimulating the production of chemokines, directing macrophage polarization, and modulating the expression of neutrophil adhesion and activation markers (Niyonsaba et al., 2013). IDR-1018, is a peptide of this class consisting of 12 *a.a*. (VRLIVAVRIWRR-NH_2_) and has been shown to enhance the anti-inflammatory response while maintaining key pro-inflammatory processes important in fighting off infection, an ability made possible by the fact that this peptide drives macrophage polarization toward an intermediate M1-M2 state (Pena et al., 2013). Other members of this class of peptide include IDR-HH2 and IDR-1002, both of which have similar immunomodulatory abilities. Antimicrobial peptides LL-37 and SET-M33 have also been shown to mediate inflammation through the reduction of pro-inflammatory cytokines, enzymes, and transduction factors (Kahlenberg and Kaplan, 2013; Brunetti et al., 2016).

One of the ways in which macrophage activation controls the immune response is through the expression/production of matrix metalloproteinases (MMPs). MMPs are a family of proteolytic enzymes which themselves are capable of modulating immune responses through the regulation of cytokines and chemokines. There are a handful of different types of MMPs all of which are capable of degrading extracellular matrix proteins and activating bioactive molecules via proteolytic cleavage or other modifications. Through the inclusion of MMP binding and cleavage sequences within the peptides that comprise a material it is possible to increase its local concentration while enhancing the proteolytic degradation of the material which can allow for its replacement with endogenous matrix and the release of small peptide fragments which can in turn modulate other cellular responses. An example, of an MMP epitope found in Type I collagen is the amino acid sequence GPQGIAG (Turk et al., 2001). The presence of such a sequence in collagen-PEG conjugates has been shown to enhance proteolytic degradation by both MMP-1 and MMP-2 (Turk et al., 2001; Patterson and Hubbell, 2010).

### Pro-Adherence Sequences

One of the key requirements of regenerative biomaterials is that they support the in-growth, attachment, and proliferation of endogenous cells. One way to ensure that cells attach to a material is to modify the material in such a way to incorporate a peptide that displays a specific binding sequence. One of the most ubiquitous and simple binding sequences are the RGD and RGDS motifs, which are prominent in adhesion proteins like fibronectin and fibrinogen but also structural proteins such as collagen and laminin (Yamada, 1991). They act as an anchoring site for a number of different α and β integrin binding receptors. The RGDS sequence has also been shown to inhibit platelet aggregation and as such displays some anti-thrombolytic activity (Samanen et al., 1991). As a polar opposite to RGD, KGD sequences have been found to disrupt cell attachment by inhibiting integrin binding (Scarborough et al., 1991). Another pro-adherence sequence derived from the adhesion protein fibronectin is PHSRN. PHSRN like RGD is an integrin cell adhesion motif, however it differs from many of the other linear cell attachment sequences in that the spatial organization of this sequence must mimic that found in fibronectin for it to be beneficial (Mardilovich et al., 2006). Also coming from fibronectin are the REDV, LDV, and KQAGDV integrin binding motifs, which have been shown to help in the anchoring of human umbilical vein endothelial cells (HUVECs) as wells as promote smooth muscle cell adhesion (Hubbell et al., 1991; Mould et al., 1991; Shin et al., 2003). Laminin derived sequences such as IKVAV and YIGSR are also important as integrin binding ligands. While YIGSR also displays some anti-cancer properties, both YIGSR and IKVAV sequences have been shown to stimulate neurite growth and have found use in the design of several therapeutic materials (Graf et al., 1987a; Tashiro et al., 1989). Structural proteins such as collagen also display some cell adhesion sequences, with the most well-described ones being derived from collagen type I and IV. As is the case with the previously mentioned pro-adherence sequences, the short DGEA, GFOGER (where O is hydroxyproline), and GFPGER sequences play a key role in integrin recognition and as such have been incorporated in a number of tissue repair strategies (Staatz et al., 1991; Knight et al., 2000).

Also important to mention is that some peptide sequences can self-assemble due to intra or inter molecular interactions to form supramolecular structures driven by Van-Der-Waals, electrostatic, hydrogen bonding, hydrophobic, and π-π stacking (see Table 1). Different kinds of self-assembly structures can be generated from the peptides depending on their structure at the nanometer scale. Peptides can assemble as (i) α-coil, (ii) β-sheet, (iii) β-hairpins, and (iv) poly-proline helix. Depending on the supramolecular structure of the peptide assembly a variety of configurations can be achieved, which include vesicles, rods, or fibers. In addition, advancements in peptide synthesis have allowed for the fabrication of peptides bearing more than one property in their sequences. Thus, for example, in Table 2, some peptide sequences contain a self-assembly portion and a second portion which acts as a bio-recognition sequences for receptors, or a lipid-like portion for vesicle formation (see Table 1).

**Table 2:** Peptide-containing biomaterials as therapeutic agents for tissue and organ repair of cornea, skin, and heart tissues.

**Peptide sequence**	**Tissue/Organ**	**Functional effect**	**Specific cell receptor**	**Delivery System**	***In vitro* or *In vivo test***	**Main findings**	**References**
CG(PKG)_4_(POG)_4_(DOG)_4_, *with O being hydroxyproline*	Cornea	Corneal implant promoting cell and nerve regeneration	–	Self-assembly	Collagenase Cell proliferation *In vivo* biocompatibility by subcutaneous implantation Corneal implantation (pigs) *In vitro* toxicology, biocompatibility, metabolic activity, live/dead, DSC	Corneal implant was compatible for transplantation showing cell and nerve regeneration	Islam et al., 2016 Jangamreddy et al., 2018
YIGSR		Promotes epithelial cell growth and neurite extension	Epithelial cells	Hydrogel	*In vitro* characterization (cell layers and thickness, nerve density, IR spectroscopy), immunohistochemistry, regeneration, corneal touch sensitivity	Overall corneal regeneration including nerve regeneration	Li et al., 2003
							
Q11 (Ac- QQKFQFQFEQQ-Am)	Skin	Wound healing in strong immune response		dermal	Wound closure, type of cell recruitment in mice with strong immune response	Immunogenic peptides do not delay healing, even in mice with heightened immune response	Vigneswaran et al., 2016
KGF–ELP		Chronic wound healing	KGF receptor	Fibrin hydrogel vehicle	Characterization (DLS, TEM), cell proliferation, full thickness wound healing	Enhanced granulation and reepithelialization	Koria et al., 2011
Pexiganan Acetate GIGKFLKKAKKFGKAFVKILKK		Antibacterial properties	Disturbs membrane permeability	Topical	MIC against gram-negative and positive bacteria, anaerobes, *in vivo* antibacterial activity, short term tolerability tests	Indication: infected diabetic foot ulcers, similar efficacy to ofloxacin	Lamb and Wiseman, 1998
HBPA (palmitoyl–AAAAGGGLRKKLGKA)		Increased angiogenesis	VEGF and FGF-2	Gel administered subcutaneously	Subcutaneous implantation, histological and morphological analysis of wound site, skinfold chamber model, *in vivo* microscopy, microcirculatory analysis	Increased angiogenesis, including *de novo* angiogenesis	Ghanaati et al., 2009
RADA16-I, [COCH3]-RADARADARADARADA-[CONH2] with EGF		Improved would healing	Keratinocytes and fibroblasts	Topical	*In vitro* human skin equivalent wound healing model, proliferation assay, apoptosis assay, Histological analysis	Epithelialization and wound healing are accelerated with EGF and RADA-16, as opposed to RADA-16 alone	Schneider et al., 2008
RADA16-GG-RGDS and RADA16-GG-FPGERGVEGPGP		Improved cell migration	Keratinocytes and fibroblasts	Hydrogel	*In vivo* analysis, including SEM of cells with SAP, cell proliferation, cell migration	Improved cellular migration	Bradshaw et al., 2014
(RADA)_4_	Heart	Self-assembling		Nanofiber	Rat MI model	Improved Angiogenesis	Dubois et al., 2008
(RADA)_4_-LRKKLGKA		Self-assembling heparin-binding sequence		Nanofiber with VEGF	Rat MI model	Improved Angiogenesis Improved Left ventricle contraction Decrease Fibrosis and Left ventricle remodeling	Guo et al., 2012
(RARADADA)_2_		Self-assembling		IFG-1 bound nanofiber with CMs	Rat MI model	Improved Cell survival Improved Left ventricle contraction Decrease cardiac remodeling	Davis et al., 2006
		Self-assembling		Dissolved in solution with MNCs	Porcine MI model	Improved Angiogenesis, Cell survival, and Left ventricle contraction. Reduced ventricular remodeling	Lin et al., 2010, 2015
		Self-assembling		Nanofiber with VEGF	Rat MI model Porcine MI model	Improved Angiogenesis Left ventricle contraction. Reduced ventricular remodeling	Lin et al., 2012
		Self-assembling		PDGF bound nanofiber	Rat MI model	Improved Angiogenesis, Cell survival and Left ventricle contraction. Reduced ventricular remodeling	Hsieh et al., 2006a,b
		Self-assembling		Dissolved in solution with ADSCs	Rat MI model	Improved Angiogenesis, Cell survival and Left ventricle contraction. Reduced ventricular remodeling	Kim et al., 2017
		Self-assembling		SDF-1 bound nanofiber	Rat MI model	Increases EPC recruitment, Angiogenesis and Left ventricle contraction	Segers et al., 2007
		Self-assembling heparin-binding sequence		Nanofiber with MSCs	Rat MI model	Increases cell survival, Angiogenesis and Left ventricle contraction	Cui et al., 2010
(RARADADA)_2_-CDDYYYGFGCNKFCRPR(Notch ligand Jagged-1)		Self-assembling Cell adhesion sequence		Hydrogel with CACs	Rat MI model	Increases cell survival and Left ventricle contraction. Decreases ventricular remodeling	Boopathy et al., 2014
AAAAGGGEIKVAV(peptide amphiphile)-YIGSR AAAAGGGEIKVAV(peptide amphiphile)-KKKKK		Self-assembling EC adhesive ligand NO producing donor		Nanofiber	N/A	Increases EPC viability and differentiation	Andukuri et al., 2013
Heparin-AAAAGGGEIKVAV(peptide amphiphile)		Self-assembling		VEGF/bFGF bound nanofiber	Mouse MI model	Increases Angiogenesis and Left ventricle contraction	Webber et al., 2010a
VVAGEGDKS		Glycosaminoglycan mimetic		Nanofiber	Rat MI model	Increases Angiogenesis and Left ventricle contraction	Rufaihah et al., 2017
AcSDKP(Thymosinβ4)		Angiogenic		Collagen-chitosan hydrogel	Rat MI model	Increases Angiogenesis and cell survival. Reduces ventricular remodeling	Chiu et al., 2012
KAFDITYVRLKF-AcSDKP(Thymosinβ4)		Proangiogenic Anti-inflammatory		Collagen hydrogel	Mouse subcutaneous implant	Increases Angiogenesis. Reduces Inflammation	Zachman et al., 2013
RGD		Cell adhesion sequence		Alginate microsphere with MSCs	Rat MI model	Improved Angiogenesis, Cell survival, and Left ventricle contraction. Reduced ventricular remodeling	Yu et al., 2010
RGD		Cell adhesion sequence		Alginate scaffold	Rat MI model	Improved Angiogenesis and Left ventricle function	Yu et al., 2009
RGDfK		Cell adhesion sequence		Alginate scaffold with MSCs	Rat MI model	Improved Angiogenesis and Left ventricle contraction	Sondermeijer et al., 2017
RGDS-AAAAGGGEIKVAV(peptide amphiphile)		Cell adhesion sequence Self-assembling		Subcutaneous injection with MNCs	Mouse	Improved Cell survival	Webber et al., 2010c
RGDSP-(RADA)_4_		Cell adhesion sequence Self-assembling		Dissolved in solution with MCSCs	Rat MI model	Improved Cell survival and Left ventricle contraction. Reduced fibrosis	Guo et al., 2010
GGGGRGDY		Cell adhesion sequence		Alginate scaffold	N/A	Improved NRVM contractility and viability	Shachar et al., 2011
GRGDS		Cell adhesion sequence		Collagen hydrogel	N/A	Improved NRVM contractility and viability	Schussler et al., 2009
QHREDGS		Cell adhesion sequence		Collagen-chitosan scaffold	N/A	Improved EC survival and tube formation	Miklas et al., 2013
		Cell adhesion sequence		Collagen-chitosan scaffold	N/A	Improved NRVM survival	Reis et al., 2012
		Cell adhesion sequence		Azidobenzoic acid-chitosan scaffold	N/A	Improved NRVM survival	Rask et al., 2010
		Cell adhesion sequence		Collagen-chitosan hydrogel	Rat MI model	Improved Cell survival and Left ventricle contraction. Reduced ventricular remodeling	Reis et al., 2015
WKYMVm		Formyl peptide receptor 2 agonist		Dissolved in solution	Mouse MI model	Improved Angiogenesis and Left ventricle contraction. Reduced fibrosis	Heo et al., 2017
KPVSLSYRCPCRFFESHPPLKWIQEYLEKALN		SDF-1a analog		Dissolved in solution	Mouse MI model	Improved Angiogenesis and Left ventricle contraction	Hiesinger et al., 2011
YPHIDSLGHWRR		78kDa Glucose-regulated protein receptor's ligand		Chitosan hydrogel	Rat MI model	Improved, Cell survival, Angiogenesis and Left ventricle contraction. Reduced ventricular remodeling	Shu et al., 2015
MHSPGAD		Stem cell recruitment		Collagen hydrogel	Mouse MI model	Improved Angiogenesis and Left ventricle contraction. Reduced fibrosis and ventricular remodeling	Zhang et al., 2019

## Applications of Peptides in Tissue Engineering and Biomaterials

The modification of materials through bioengineering techniques has given rise to a promising route for the generation of synthetic and hybrid materials which not only display biological function and compatibility, but are also capable of controlling cellular microenvironment. The field of tissue engineering is continuously evolving and improving, changing the way scientists and engineers treat damaged tissues (Chen and Liu, 2016). One of the most important aspects of tissue engineering is the design of materials that are biocompatible and capable of interacting with cells and the host environment to promote healing (Girotti et al., 2004; Chen and Liu, 2016). To this end, a number of matrices have been developed for applications that range from tissue replacement and repair to drug delivery (Girotti et al., 2004; Chow et al., 2011; Chen and Liu, 2016). Peptides are increasingly being incorporated or self-assembled into matrices to enhance cell signaling and the bioactivity, improve drug delivery, and provide antibacterial properties amongst many other applications (Girotti et al., 2004; Chow et al., 2011; Miotto et al., 2015). In this section, we will briefly review some representative examples of peptide-based approaches for regenerative therapies in heart, skin, and cornea (Girotti et al., 2004; Chattopadhyay and Raines, 2014; Rodríguez-Cabello et al., 2018). In selecting literature, we have limited our search to articles that contain *in vivo* assessments for the materials, see Table 2.

### Peptides Sequences in Cornea and Skin Therapeutics

Collagen- and elastin-like peptides are commonly used peptides in skin and cornea tissue repair (Chattopadhyay and Raines, 2014; Rodríguez-Cabello et al., 2018). Collagen is the most abundant protein in the extracellular matrix and commonly used in biomaterials (Chattopadhyay and Raines, 2014; Tanrikulu et al., 2016). Full-length human collagen is complicated to synthesize, as it requires significant post-transcriptional modifications and it is not soluble in most buffers, making it difficult to study (Koide, 2005; Tanrikulu et al., 2016). However, short collagen-mimetic peptide sequences are being used to mimic full-length collagen, by incorporating important peptide sequences at a fraction of the length (Tanrikulu et al., 2016). These collagen mimetic sequences typically require a glycine residue present in every third position and contain many proline and hydroxyproline repeats (Chattopadhyay and Raines, 2014; Tanrikulu et al., 2016). These sequences form left-handed polyProline II helix chains, which then self-assemble in groups of three to produce a right-handed superhelix (Fields, 2010; Chattopadhyay and Raines, 2014; Tanrikulu et al., 2016). The peptide sequence (PKG)_4_(POG)_4_(DOG)_4_ has also been designed to self-assemble as a collagen mimetic peptide (O'leary et al., 2011). The N-terminus of this self-assembling peptide was then modified to contain a glycine spacer and terminal cysteine (CG-linker). The addition of a terminal cysteine allowed for the attachment of the peptide to an 8-arm PEG polymer via maleimide chemistry. The application of this new collagen mimetic peptide-hybrid polymer as solid implant resulted in transparent and well-shaped corneas with the deposition of new collagen and the infiltration of stromal cells after 12 months of implantation in porcine model (Islam et al., 2016). An improvement in the formulation was achieved through the addition of the molecule 2-methacryloyloxyethyl phosphorylcholine (MPC), which has been shown to reduce inflammation and improve hydrogel bio-compatibility (Jangamreddy et al., 2018). In terms of recovery after 12 months, the same epithelium, stromal and nerve recovery was found between the improved formulation and a cornea model graft made from Type III Recombinant Human Collagen.

The laminin adhesion pentapeptide motif, YIGSR, has also been grafted onto biosynthetic corneas comprised of hydrated collagen and *N*-isopropylacrylamide copolymers, and tested in Yucatan micropigs (Li et al., 2003). The materials were 5.5 mm in diameter and 200 μm think and implanted via lamellar keratoplasty. After 6 weeks, the implants were able to demonstrate successful regeneration of the host corneal epithelium, stroma, and nerves. In contrast, no nerve regeneration was observed in control eyes which received allografts, during the experimental period (Li et al., 2003).

Peptides have also been functionalized in ways which allow them to be tethered to nanoparticles to generate biomimetic platforms, alter physical properties and cellular interactions, or allow for their incorporation into fibrils or hydrogels for various application (Chattopadhyay and Raines, 2014; Chen and Liu, 2016; Rodríguez-Cabello et al., 2018).

Elastin-like peptides (ELPs) have shown to be extremely useful in tissue engineering, due to their elastic properties, which help them mimic the physical properties of a number of different tissues and organs (Rodríguez-Cabello et al., 2018). While the abundance of elastin in the human body is low (2–4% of dry weight of skin) it plays an important part in the mechanical strength and support of skin and has also been demonstrated to be involved in cell signaling (Rodríguez-Cabello et al., 2018). ELPs are typically derived from the pentapeptide sequence of elastin (VPGXG), where X can be any amino acid (Urry et al., 1981; Urry, 1988; Girotti et al., 2004; Rodríguez-Cabello et al., 2018). This sequence maintains its elastomeric properties when it is crosslinked (Urry et al., 1981; Girotti et al., 2004). It has been suggested that the human body cannot discern ELPs from endogenous elastin and ELP matrices show similar mechanical properties as endogenous elastin, which allows the body to use the scaffold to rebuild the natural ECM (Girotti et al., 2004).

Peptides such as Q11 and RADA-16, have also been incorporated into biomaterials and used in tissue engineering (Vigneswaran et al., 2016). RADA-16 with EGF has shown to improve cell mobility in the skin, which can result in improved wound healing, especially in non-healing wounds (Schneider et al., 2008; Bradshaw et al., 2014). Lastly, wound healing antimicrobial peptides (AMPs) have also been used in applications of non-healing infected wounds, such as diabetic foot ulcers. These peptides prevent infection, reduce inflammatory response, and promote cell proliferation and migration (Mangoni et al., 2016; Gomes et al., 2017). AMPs have a wide range of amino acid sequences, however they are generally composed of an amphipathic structure, which contains a high prevalence of basic residues (Mangoni et al., 2016). In human skin, AMPs are synthesized and stored by keratinocytes in the granular layer (Mangoni et al., 2016).

### Applications in the Heart

Myocardial infarction (MI) is a leading cause of death globally, and can ultimately lead to heart failure (World Health Organization, 2017). In order to be effective peptide-based therapeutics need to be resistant to local proteases and retained long enough to exert the desired effect in the myocardium. The employed self-assembling peptides are typically comprised of alternating hydrophilic and hydrophobic amino acids (Zhang, 2003), which on exposure to physiological osmolality and pH, rapidly assemble into nanofibrous structures that can be injected into the myocardium to form 3D microenvironments (Zhang et al., 1993; Davis et al., 2005). Such therapy has shown promise in the treatment of infarcted myocardium. The RADA class of ionic self-complementary peptide is one of the first generations of self-assembling peptide and the most intensively studied for applications in MI, as it is commercially available (Dubois et al., 2008). When delivered with platelet-derived growth factor (PDGF), the self-assembling nanofibers fabricated from the RADA sequence decreased infarct size and improved cardiac function in a rat MI model (Hsieh et al., 2006a,b). Despite cardiac-specific overexpression of several members of the PDGF family and the fact that it has been reported to induce fibroblast overgrowth and cardiac fibrosis (Ponten et al., 2005), this study demonstrated that PDGF conjugated to the self-assembling nanofibers actually reduced cardiac fibrosis, suggesting a well-controlled release of PDGF. When combined with VEGF, the RADA-derived nanofibrous hydrogels were also shown to improve angiogenesis and cardiac performance in rat and porcine MI models (Lin et al., 2012). The RADA sequence has also been used in combination with cell therapies. For example, injection of a RADA derived hydrogel into a porcine MI model with bone marrow mononuclear cells (MNCs) increased cell retention about 8-fold and improve the cardiac function at 1 month post-MI (Lin et al., 2010, 2015). Similarly, human adipose-derived stromal cells (ADSCs) with fibroblast growth factor (FGF)-immobilized within a RADA hydrogel were injected into a rat MI heart, and demonstrated to promote angiogenesis and improve cardiac contraction (Kim et al., 2017). Likewise, tethering of insulin-like growth factor-1 (IGF-1) to self-assembling peptides increased survival of transplanted neonatal rat cardiomyocytes in a rat MI model (Davis et al., 2006). Cell mediated therapies are also enhanced by well-controlled release of some types of chemokines. Stromal cell-derived factor-1 (SDF-1) was combined to RADA nanofibers, and was demonstrated to improve cardiac function via recruitment of endothelial progenitor cells (EPCs) (Segers et al., 2007). Of note, is the fact that the SDF-1 has also been attached to a 6-amino acid sequence susceptible to MMP-2 cleavage to achieve “smart release” of the chemokine at the site of infarction, albeit showing no additional effect *in vivo* (Segers et al., 2007).

Self-assembled peptide amphiphiles have emerged as versatile biomaterials (Beniash et al., 2005). The amphiphilicity of the peptides allows for self-assembly in aqueous media, eliminating the necessity of organic solvents and as such broadens their applicability. To improve cell retention, a peptide amphiphile scaffold was combined with RGDS, and delivered with MNCs subcutaneously (Webber et al., 2010c). The incorporation of RGDS improved retention and proliferation of the cells *in vivo*, along with enhancing endothelial marker expression *in vitro*. Likewise, heparin-binding peptide amphiphile (HBPA) was developed and assessed as a biomaterial for MI therapies, which was designed to mimic natural heparin-binding proteins and enable binding to a variety of proteins, increasing cellular recognition of these factors (Rajangam et al., 2006). When combined with VEGF or FGF, HBPA demonstrated improved angiogenesis and heart contractility in mouse (Webber et al., 2010a). Heparin is known to preserve growth factors in their active form by protecting them from proteolysis, and enhancing the affinity to their respective receptors, enabling consistent release of growth factors (Zhou et al., 2004); however, the use of heparin could trigger immune reactions due to its animal origin. To overcome this limitation, synthetic glycosaminoglycan (GAG) mimetic peptide nanofiber scaffolds were developed and assessed *in vivo* (Rufaihah et al., 2017). The GAG scaffolds induced neovascularization in the infarcted myocardium, along with increased VEGF expression and recruitment of vascular cells, which lead to significant improvements in cardiac performance.

Given the “hostile” environment within the infarcted heart, another approach has been to deliver soluble peptides within polymeric scaffolds to mimic extracellular matrix degradation products, which can act in a cytokine fashion (Zachman et al., 2013). The pro-angiogenic laminin-derived C16 and the anti-inflammatory thymosin β4-derived Ac-SDKP loaded in collagen hydrogels of scaffolds has shown to up-regulate the angiogenic response in subcutaneous implantation, while down-regulating inflammation, thus holding promise as a strategy for addressing ischemia and inflammation post-MI. Thymosin β4 has also been successfully incorporated into collagen-chitosan hydrogels for release in the heart post-MI, resulting in superior vascular growth and myocardial repair compared to unmodified hydrogels (Chiu et al., 2012).

### Modification for Combination Therapies With Cells

Some large extracellular matrix (ECM) molecules, such as collagen and fibronectin, have multiple peptide sequences that are recognized by cells and induce multiple regenerative responses. To tackle the issue of poor retention and survival of reparative cellular components for MI, mimics of the nanotopographical cues of native ECM have been used to improve integration, proliferation and differentiation. The RGD sequence has been identified as the major cell-binding domain in fibronectin (Ruoslahti and Pierschbacher, 1987), and is able to act as ligands for the integrins αvβ5, αvβ3, and α5β1, which are expressed by cardiomyocytes (Ross Robert and Borg Thomas, 2001; Brancaccio et al., 2006). Functionalization of materials with the RGD motif may exert advantageous properties to the regenerating myocardium via better adhesion and cell integration. RGD incorporation into collagen and alginate scaffolds has been shown to improve cardiomyocyte contractility and viability (Schussler et al., 2009; Shachar et al., 2011). An RGD-alginate system was also able to improve vascular endothelial cell adhesion and proliferation, and increase blood vessel formation *in vivo* (Yu et al., 2009). When applied as microspheres encapsulating mesenchymal stem cells (MSCs); the RGD-alginate combination improved cell retention at the site of injection, in addition to enhanced arteriole formation in a rat MI model (Yu et al., 2010). Similarly, alginate scaffolds modified with a cyclic RGDfK-peptide, which is protease resistant and displays high affinity to cellular integrins, improved survival of transplanted MSCs and promoted angiogenesis in a rat MI model (Sondermeijer et al., 2017). RGDSP is also an adhesion sequence, which promotes cell adhesion and stimulates integrins relevant to early cardiac development (Kraehenbuehl et al., 2008). When combined with self-assembling peptide RADA16, the RGDSP scaffolds elicited protective effects for marrow-derived cardiac stem cells, which were isolated from MSCs and identified as c-kit, Nkx2.5, and GATA4 positive populations, and improved the cardiac function of post-MI rats via enhanced cardiac differentiation (Guo et al., 2010). RGDSP showed fibrous structure with nanometer diameters when assembled with RADA16, providing 3-dimensional scaffolds and presumably being beneficial to the microenvironment for the growth of transplanted cells. The YIGSR sequence (laminin-derived) is another example of ECM-derived peptide that has been investigated as functional additive to enhance cell therapies (Boateng et al., 2005). In one study, YIGSR was immobilized into a self-assembled peptide amphiphile in combination with a nitric oxide donor polylysine sequence (KKKKK) (Andukuri et al., 2013). The combination of these peptides was superior in capturing EPCs and inducing their differentiation into endothelial cells. QHREDGS is also a type of ECM-derived peptide, based on the fibrinogen-like domain of angiopoietin-1 (Rask et al., 2010; Miklas et al., 2013). Due to the homologous nature of the integrin ligands, QHREDGS sequence reportedly has a dual protective effect for both cardiomyocytes and endothelial cells *in vitro* (Reis et al., 2012). In rat MI model, QHREDGS incorporated within a collagen-chitosan hydrogel was demonstrated to improve cardiac function along with cardiac cell recruitment via β1-integrin (Reis et al., 2015). Although this data is promising in terms of cell recruitment to the site of treatment, the provoked downstream signaling may not be the same as that of native matrix possibly due to the other components contained within ECM proteins or structural differences. In an *in vitro* study, myocytes cultured with RGD and YIGSR peptides showed lower expression of focal adhesion kinase (FAK), a part of mechano-transduction pathways, even though the adhesion of the cells was comparable to the native proteins, fibronectin and laminin, and the β1-integrin expression levels were unchanged (Boateng et al., 2005).

Other peptide ligands which are fundamental to specific cell types have also been investigated. For instance, due to the fact that Notch signaling has been shown to promote cardiac progenitor cell (CPC) mediated cardiac repair (Boni et al., 2008), RADA self-assembling peptides have been functionalized with a peptide mimic of the Notch1 ligand Jagged1 and demonstrated to have therapeutic benefit when transplanted with CPCs by improving acute retention and ameliorating the cardiac remodeling in a rat MI model (Boopathy et al., 2014). Development of biomaterials which are capable of modulating signaling pathways critical for endogenous cell types such as NOTCH1 is of great importance as these cells are endogenously present in niches of defined composition and exert reparative effects depending on environmental cues following injury, aging or disease (Sanada et al., 2014). Circulating angiogenic cells (CACs) are another promising candidate of cell therapy for MI, playing essential roles in angiogenesis and myocardial regeneration. Formyl peptide receptor 2 (FPR2), belonging to the G protein-coupled receptor family, has been suggested to stimulate and promote chemotaxis of monocytic cell lines, neutrophils, and B lymphocytes (Gavins, 2010). WKYMVm, a synthetic hexapeptide with strong affinity to FRP2 was injected to post-MI mice, and demonstrated to enhance the mobilization of CACs from the bone marrow, this resulted in myocardial protection from apoptosis with increased vascular density and preservation of cardiac function (Heo et al., 2017). Likewise, stromal cell–derived factor-1 (SDF-1) is one of the key regulators of hematopoietic stem cells, and shown to effect proliferation and mobilization of EPCs, one of the major population of CACs, to induce vasculogenesis and to be significantly upregulated in myocardial ischemia (Pillarisetti and Gupta, 2001). However, exogenous SDF is quickly degraded by multiple proteases (Sierra et al., 2004). To overcome this limitation, a polypeptide analog was engineered and demonstrated enhanced physiological ability to induce EPC migration and improved ventricular performance compared with native SDF (Hiesinger et al., 2011). In another study, RoY, a 12 amino-acid synthetic peptide specifically binding to the 78 kDa glucose-regulated protein (GRP78) receptor, which is largely expressed on vascular endothelial cells under hypoxia, was conjugated to a thermosensitive chitosan chloride hydrogel. The material induced angiogenic activity and attenuated myocardial injury in a rat MI model (Shu et al., 2015). Histone deacetylase 7 (HDAC7)-derived- phosphorylated 7-amino-acid peptide has also been successfully incorporated into collagen hydrogels for release in the heart post-MI, resulting in superior vascular growth and myocardial repair via enhanced stem cell antigen-1 (Sca-1) positive stem cell recruitment and differentiation (Zhang et al., 2019). Although peptide-based strategies allow for control over cell adhesion, signal localization and cytokine release, the peptides are often highly ubiquitous and not specific to particular cell types or signaling pathways. Further investigations are required before these therapeutic materials are ready for clinical application.

## Conclusions and Outlook

As the field looks to develop clinically translatable biomimetic materials for tissue regeneration, it is evident that peptide-based biomaterials have the ability to give rise to therapies which will not only provide improved quality of life, but also solve current problems associated with the xenogeneic nature of animal derived materials and the high cost of recombinantly prepared proteins. Due to recent advancements in SPPS and a better understanding of the structure-function relationship of peptides and proteins in complex biological settings, it is becoming more feasible to design targeted biomaterials capable of eliciting a desired response or enhanced biocompatibility. These short mimetic peptides are also typically more processable than their full length analogs and as such simpler to modify with a variety of different functionalities which could impart beneficial properties such as enhanced solubility, simple one step tethering to polymeric backbones, or stimuli responsiveness (pH, light, temperature, etc.). Given the complexity of the wound healing process, as we learn more about the factors determining tissue regeneration, it is likely that we will begin to see an increase in the development of combinatorial approaches and the design of materials consisting of numerous different structural and sequence based peptide mimics. While such complex materials are currently difficult to design, as predictive models improve and large bioactive peptide databases become available this task will be greatly simplified.

## Author Contributions

All authors listed have made a substantial, direct and intellectual contribution to the work, and approved it for publication.

### Conflict of Interest Statement

The authors declare that the research was conducted in the absence of any commercial or financial relationships that could be construed as a potential conflict of interest.
